# Effect of Young Plasma Therapy on Cognition, Oxidative Stress, miRNA-134, BDNF, CREB, and SIRT-1 Expressions and Neuronal Survey in the Hippocampus of Aged Ovariectomized Rats with Alzheimer’s

**DOI:** 10.3390/brainsci14070656

**Published:** 2024-06-28

**Authors:** Parisa Habibi, Siamak Shahidi, Maryam Khajvand-Abedini, Zahra Shahabi, Nasser Ahmadiasl, Mohammad Reza Alipour, Mahdi Ramezani, Alireza Komaki

**Affiliations:** 1 Neurophysiology Research Center, Hamadan University of Medical Sciences, Hamadan 651783873, Iran; dr.habibi2007@gmail.com (P.H.);; 2 Department of Physiology, School of Medicine, Tehran University of Medical Sciences, Tehran 1461884513, Iran; 3 Department of Clinical Biochemistry, Faculty of Medicine, Hamadan University of Medical Sciences, Hamadan 651783873, Iran; 4 Neurosciences Research Center, Tabriz University of Medical Sciences, Tabriz 5166616471, Iran; 5 Stem Cell Research Center, Tabriz University of Medical Sciences, Tabriz 5166616471, Iran; 6 Department of Anatomy, School of Medicine, Hamadan University of Medical Sciences, Hamadan 651783873, Iran

**Keywords:** ovariectomy, Alzheimer, young plasma, estrogen, miR-134a

## Abstract

Menopause may increase the risk of Alzheimer’s disease (AD) dementia. This study aimed to use young plasma therapy (YPT) to improve dementia caused by AD in aged ovariectomized rats. Female Wistar rats were used in the following groups: (a) young (CY) (180–200 g, 2–3 months, n = 10) and (b) old groups (250–350 g, 22–24 months, n = 60). The old rats were randomly assigned to six sub-groups: (1) control, (2) sham, (3) ovariectomized group (OVX), (4) OVX + Alzheimer disease (OVX + AD), (5) OVX + AD+ 17β-Estradiol (OVX + AD + E), and (6) OVX + AD + young plasma (OVX + AD + YP). Cognitive behaviors were evaluated using NOR, MWM, and PAL tests. MiR-134a, SIRT-1, CREB, and BDNF expressions were measured using real-time PCR and western blot, respectively. Oxidative stress in hippocampal tissue was assayed using ELISA kits. OVX and AD caused significant cognitive impairment (*p <* 0.001), up-regulated miR-134a (*p <* 0.001), down-regulated SIRT-1, CREB, and BDNF protein expression (*p <* 0.001), and decreased antioxidant marker levels (*p <* 0.001) compared to the sham group. YPT significantly restored miR-134a (*p <* 0.001), SIRT-1 (*p <* 0.001), CREB (*p <* 0.001), and BDNF (*p <* 0.001) protein expression in OVX + AD rats. YPT, as much as or more than estrogen therapy (ERT), significantly improved oxidative stress and down-regulated miR-134a expression and the up-regulation of SIRT-1, CREB, and BDNF proteins in OVX + AD rats (*p <* 0.001). YPT significantly improved histological alteration compared to the OVX + AD group (*p <* 0.001). As a non-pharmacological treatment, YPT can improve the expression of miR-134a and SIRT-1, CREB, and BDNF proteins as much as or more than estrogen therapy, ameliorating AD-induced dementia in aged OVX rats.

## 1. Introduction

Alzheimer’s disease is one of the neurodegenerative diseases associated with amyloid (Aβ) deposition in senile plaques, apoptosis of cerebral cortex and hippocampal neurons, and synaptic loss [1]. Alzheimer’s disease is more common in postmenopausal women and is characterized by progressive impairment of memory and other cognitive functions [2]. The increase in the deposition of Aβ plaques in the brain of postmenopausal women probably indicates the relationship between estrogen deficiency and neurological disorders in middle-aged women [3]. Indeed, estrogen plays an important role in regulating brain development, brain function, and behavior [4].

In patients with AD, the brain is exposed to decreased antioxidant defense and increased production of reactive oxygen species (ROS) [5].

Sirtuins (SIRT) are a family of NAD (+)-dependent class III histone deacetylases. At this time, seven sirtuin homologs are known in mammals, from sirtuin 1 (SIRT-1) to sirtuin 7 (SIRT-7), which have different vital functions. SIRT-1 and SIRT-6 regulate aging-related cellular stresses via several major pathways, and thus, SIRT modulators show therapeutic potential for the treatment of age-related diseases [6]. During aging, SIRT-1 is responsible for the maintenance of neural systems and behavior, including the modulation of synaptic plasticity and memory processes, and the deficiency of SIRT expression in the hippocampal neurons weakens cognitive function, as well as recent memory and spatial learning [7].

Among all neurotrophins, brain-derived neurotrophic factor (BDNF) stands out for its high level of expression in the brain and its potent effects on synapses [8]. The synergistic interactions between neuronal activity and synaptic plasticity by BDNF make it an ideal and essential regulator of cellular processes that underlie cognition and other complex behaviors such as learning and memory. Investigations suggest that deficits in BDNF signaling contribute to the pathogenesis of several major diseases and disorders, such as Huntington’s disease, Alzheimer’s disease, and depression [9].

The hippocampus, one of the brain regions involved in memory consolidation, is particularly sensitive to oxidative stress that leads to neuronal death [1]. Decreased expression of some brain factors in the hippocampus, such as brain-derived neurotrophic factor (BDNF), cAMP-responsive element binding protein (CREB), and sirtuin protein-1 (SIRT-1) are involved in these types of vulnerability [10].

MicroRNAs, small non-coding RNAs, participate in various features of hippocampus physiology, such as growth, neurogenesis, and synapse establishment. Moreover, dysregulation of microRNAs contributes to the pathology of numerous hippocampus-related disorders including Alzheimer’s disease, schizophrenia, and epilepsy [11]. miR-134, miR-146-5p, miR-8-5p, miR-124, miR-138, miR-184, and miR-137 are the most studied microRNAs in the hippocampus [12]. Ceren Eyileten, in the study in 2021, showed that the up-regulation of miR-134a can target the BDNF gene and affect expressions of that [13]. The NAD-dependent deacetylase SIRT-1 is vital for learning, and this function requires interaction with brain-specific miR-134. The transcriptional regulation of miR-134 is suppressed by SIRT-1, and miR-134 expression is up-regulated in SIRT-1-deficient mice. Overexpression of miR-134 regulates negatively memory formation in rodent hippocampus through translational repression of CREB mRNA [14].

In this regard, the neuroprotective effects of estrogen, such as preserving hippocampal function and reducing Aβ accumulation in hormone replacement therapy (HRT), have been studied in animal models [15]. However, some studies have not confirmed the beneficial effects of estrogen therapy on cognitive impairment [16]. Moreover, HRT has been implicated in causing side effects such as cancer and cardiovascular disorders and has even aggravated the severity of dementia [17]. Therefore, studies on the mechanisms of estrogen’s effects on memory in postmenopausal women are still ongoing [18].

In the last decade, parabiosis has been used in age-related disease studies [19]. Parabiosis is a technique to share circulatory systems and factors between animals (young to young or old to old (isochronic) and young to old (heterochronic) [20]. In 2020, Ashapkin et al. showed that the parabiosis method and some factors in the circulation of young mice can stimulate angiogenesis resulting from increased brain neurogenesis and olfactory strength in old mice [21]. Moreover, circulating factors in young blood can reverse age-related cognitive deficits by rejuvenating stem cells in the spinal cord and brain of aged rodents [18]. Young blood plasma improves tau and Aβ pathologies and increases cognitive function in 3×Tg-AD mice [22]. One study in 2024 revealed that small extracellular vesicles from young plasma reversed deteriorating changes and age-related dysfunction by motivating PGC-1α expression and increasing mitochondrial energy metabolism [23]. However, the role of young rat plasma in the reconstruction of aged female rat brain neurons and the rejuvenation of age-related cognitive processes remains unknown. Therefore, in the present research, the effects of young plasma therapy (YPT) compared to estrogen therapy on cognitive disorders, oxidative stress, miR-134a expression, and the expression of some of its target proteins such as BDNF, CREB, and SIRT-1 were studied in the hippocampus of an Alzheimer’s disease model in aged ovariectomized rats.

## 2. Materials and Methods

### 2.1. Experimental Design

**Figure 1:**
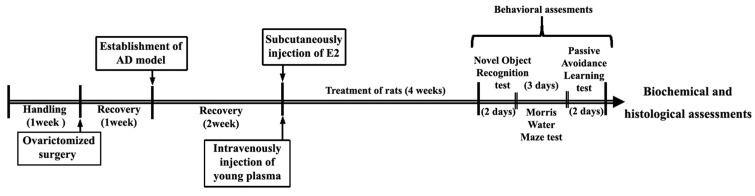
Schematic diagram of timeline and experimental design.

A total of 60 female Wistar rats, old (250–350 g, 22–24 months in 6 sub-groups (n = 10 in each group) and young (180–220 g, 2–3 months, in a single group, n = 10), were obtained from the Hamadan University of Medical Sciences (UMSHA) Animal Care and Breeding Center. The animals were kept in standard physical conditions under a 12-hour light/dark period (light on 07:00 am) with ad libitum access to rodent food and tap water. Experiments were performed from 08:00 am to 04:00 pm. The local ethics committee at UMSHA approved (IR.UMSHA.REC.1397.638) the animal care, procedures, and surgery protocols according to the National Institute of Health (NIH). For plasma collection, 30 young rats (180–220 g, 2–3 months, 15 males and 15 females) were also purchased from the same animal breeding center. The animals were randomly divided into seven experimental groups as follows:

1. CY: Young control group (female, 2–3 months old) without any surgery or treatment. The young rats with healthy status were used for comparison with old control rats.
2. CO: Old control group (female, 20–24 months old); without any surgery or treatment.
3. Sham: The old female rats received distilled water by injection into the intracerebroventricular (ICV) as Aβ_1-42_ solvent and underwent bilateral ovariectomy (OVX) without ovarian removal.
4. OVX: Old female rats underwent bilateral ovariectomy without treatment [24].
5. OVX + AD: The old female rats underwent conditions like group OVX with the induction of the AD model with Aβ_1-42_ without treatment.
6. OVX + AD + E: The old female rats underwent conditions like group OVX + AD and received 17β-estradiol (E2) (30 mg/kg, five days/week, subcutaneously; Bayer HealthCare Pharmaceuticals, Berlin, Germany) for four weeks [25].
7. OVX + AD + YP: The old rats underwent conditions like group OVX + AD and received young plasma intravenously through the tail vein with 1 mL of plasma three times a week for four weeks [26]. The experimental timeline is shown in Figure 1.

At the end of behavioral tests, each group was divided into 2 sub-groups as follows:

1. For biochemical (oxidative stress and gene expression, n = 6).
2. Histological evaluating (n = 4).

### 2.2. Ovariectomy Surgery

Briefly, after the animals were anesthetized with a combination of ketamine/xylazine (70/7 mg/kg, IP), a small incision was made on either side of the dorsal region of the rat body and the ovaries were removed bilaterally, followed by the enclosure of the fallopian tubes with tight ligatures. Operated animals had a 7-day post-surgery recovery period. Only the surgical incision was performed without ovarian resection in the sham group [27]

### 2.3. Establishment of AD Model

To establish AD in anesthetized OVX rats, using a stereotaxic apparatus and a 20 microliters Hamilton syringe, Aβ_1-42_ (5 μg/5 μL/rat, dissolved in distilled water (Sigma Aldrich, St. Louis, MO, USA)) was infused into the lateral ventricles. E2 and YP treatments were started via the tail vein 24 h after AD induction [28].

### 2.4. Plasma Collection

To prepare plasma, blood was withdrawn via the inferior vena cava from anesthetized young (2–3 months old) rats. Plasma was obtained from the blood with centrifugation at 1000× *g* for 10 min with sodium citrate. Plasma collected from male and female young rats was mixed in a 1:1 (*v*/*v*). Plasma aliquots were stored at −80 °C until use [29].

### 2.5. Behavioral Tests

The aged menopausal female rats with AD or without AD were disturbed in many cognitive behaviors. Therefore, in this study, we investigated some cognitive behavioral disorders, such as novel objective recognition, spatial learning and memory, and passive avoidance memory.

### 2.6. Novel Object Recognition (NOR)

The NOR test was used for the evaluation of working memory and the identification of a familiar object compared to a new object and is among the most commonly used behavioral tests for rats. The device used was similar to an open field (OF) device.

The experiment was conducted in three stages. In the first stage, each rat was given 10 min to explore an empty box for habituation. In the second stage, after 24 h, each rat was presented with two similar objects, and then in the third stage (after 48 h), one of the two objects was replaced by a new object. The amount of time taken to differentiate the new object provides an index of recognition memory (DI) and was calculated using the following formula [30,31]:

$$\mathrm{DI}=\frac{exploration\;time\;of\;the\;novel\;object}{total\;exploration\;time}(\times 100)$$

### 2.7. Morris Water Maze (MWM) Test

Spatial memory was assessed as previously described by Babri et al. [32]. A black circular pool (diameter, 180 cm; height, 60 cm) was filled with water (22 ± 2 °C) to a depth of 40 cm. Four quadrants on the pool were designated as north-west (NW), north-east (NE), south-east (SE), and south-west (SW) and positioned in a lit room with many visual cues. The Perspex platform (12 cm diameter) was 1.5 cm below or above the water’s surface throughout spatial learning or the observable session. The rats were trained in two consecutive blocks. Each block was contained within four trials (90 s), with different starting places equally dispersed between the first and last blocks. On day 3, each rat performed a 60-s probe trial in which the platform was removed from the pool. A camcorder (Nikon Corporation, Japan) was used to track and record escape latency on the unseen platform, swimming path length, and swimming speed [33].

### 2.8. Passive Avoidance Learning (PAL) Test

The PAL test evaluated passive avoidance memory retention deficits. The device employed in this test was made up of two chambers (dark and light) separated by a trapdoor. The test was performed in three phases. In phase one (familiar with the device), each rat was placed in the bright chamber for 60 s of familiarization while the guillotine door was opened and the animal was allowed to explore all device environments to discount the stress during the experiment. In phase two (acquisition phase), the step-through latency in the first acquisition trial (initial latency; ITL or STLa) was recorded after retesting. After the rat entered the dark chamber, the trapdoor was closed and an electric foot shock (50 Hz, 1.5 mA) was delivered to its paws for 1.5 s. After 10, the rat exited the device and was temporarily transferred to a cage. After 2 min, the above process was repeated; the rat received shock again if it went in the dark (the shocking number was recorded). If the rat did not enter the dark chamber within 120 s, passive avoidance learning was effective. The following day, the third phase (retention phase) was performed. The animals were located in the light chamber; the time spent in the dark chamber (TDC) and the step-through latency at which the animal went in the dark space lacking any electric shock (STLr) were recorded for 300 s [30].

### 2.9. Tissue Sampling

At the end of the behavioral assessments, the animals were deeply and irreversibly anesthetized with a mixture of ketamine and xylazine (100:10 mg/kg) [34]. Four animals in each group were designed for transcardiac perfusion and histopathological evaluation. Six animals from each group were used for the biochemical and molecular investigations. Similarly, after eliminating hippocampal tissues from the skull, they were rinsed with ice-cold saline, immediately frozen in liquid nitrogen, and stored at −80 °C until studied.

### 2.10. Antioxidants/Oxidants Markers Assay

A total of 40 mg of the hippocampus tissue was made uniform in a lysis buffer (10 mM (4-(2-hydroxyethyl)-1-piperazine ethane sulfonic acid), 10 mM KCl, 1.5 mM MgCl2, 1 mM EDTA, 0.1% Triton X100, and protease inhibitor cocktail, pH = 7.9) and centrifuged at 10,000× *g* for 15 min at 4 °C. The supernatant was stored at −70 °C. The tissue protein content was measured using the Bradford technique via bovine serum albumin (BSA) as a standard. Total antioxidant capacity (TAC) was measured with the ferric-reducing antioxidant power examination (FRAP) [35]. The total oxidant status (TOS) was evaluated by measuring the ability of the sample to oxidize Fe II with xylenol orange [36]. The ratio of TOS to TAC was used to analyze the oxidative stress index (OSI). The total thiol groups (TTG) and malondialdehyde (MDA), an index of lipid peroxidation, were analyzed using a Kiazist ELISA kit affording to the manufacturer’s orders (Kiazist, Tehran, Iran).

### 2.11. Western Blot Investigation

Radioimmunoprecipitation assay (RIPA) buffer comprising protease inhibitors was used to make hippocampal homogenates. The bicinchoninic acid procedure was used to evaluate total protein content. Sulfate–polyacrylamide gel electrophoresis (SDS-PAGE) was used to fractionate tissue homogenates, which were then transferred to nitrocellulose membranes. After blocking, the primary antibodies, BDNF (108319, Abcam, Boston, MA, USA) and SIRT-1, showed phosphorylation, prompting CREB (p-CREB), CREB, and GAPDH to be used as load controls (sc-74504, sc-7978, sc-377154, and sc-32233, respectively; Santa Cruz Biotechnology, California, Santa Cruz, USA). Density standards were determined using ImageJ computer software [37].

### 2.12. MiR-134a Expression Measuring

Tissue RNA was isolated using Roche TriPure reagent (# 116671657001; Roche, Mannheim, Germany). The total RNA was reverse transcribed into cDNA using a Prime Script RT reagent kit (TaKaRa Biotechnology, Shiga, Japan). U6 and miR-134a expression were measured with real-time qPCR using SYBR Green master mix (Amplicon, Odense, Denmark) in a LightCycler_96 instrument (Roche Life Science Deutschland GmbH, Sandhofer, Mannheim, Germany). The primer sequences were located as follows: 5′- GTGTGAGGTGTGACTGGTTG-3′ for miR-134a and 5ʹ -TTCGTGAAGCGTTCCATATTTT-3ʹ for housekeeping gene U6. The relation expression analysis of miR-134a was achieved using the 2^−ΔΔCt^ method [38].

### 2.13. Histological Evaluations

At the end of all behavioral tests, rats (n = 4) were anesthetized and transcardiac perfusion was performed with ice-cold 4% paraformaldehyde (phosphate-buffered). The brains of rats were carefully removed from the skull, fixed in 4% paraformaldehyde for 48 h, and embedded in paraffin. The paraffin sections were stained with toluidine blue (Merck, Darmstadt, Germany), and the Nissl bodies were stained blue-purple. Finally, the CA1 region of the hippocampus in the stained units was investigated under a light microscope (Olympus, Tokyo, Japan) for the neuronal survey.

### 2.14. Statistical Analysis

The data are shown as mean ± SEM. After establishing the normal distribution of data, all statistical analyses were performed with a repeated measures one-way ANOVA and Tukey’s post hoc tests. Alterations were considered statistically significant at *p* < 0.05.

## 3. Results

### 3.1. Novel Object Recognition (NOR)

**Figure 2:**
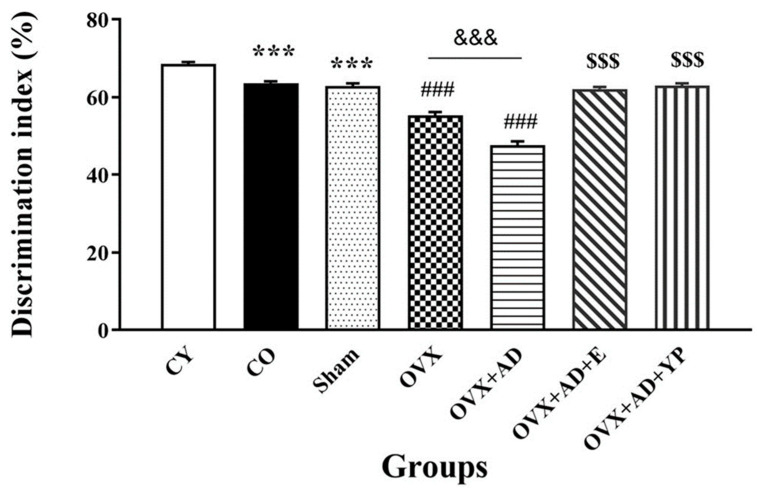
Discrimination index (DI) of all experienced groups in novel object recognition (NOR) test. Data shown as mean ± SEM and analyzed using one-way ANOVA with Tukey’s post hoc tests (n = 10), respectively. *** *p <* 0.001 vs. CY group, ### *p <* 0.001 vs. CO. and sham groups. $$$ *p <* 0.001 vs. OVX + AD groups. &&& *p <* 0.001 vs. OVX. CY; control young, CO; control old, OVX; ovariectomized, OVX + AD; ovariectomized with Alzheimer’s disease, OVX + AD + E; ovariectomized by Alzheimer’s disease treated with estradiol, OVX + AD + YP; ovariectomized with Alzheimer’s treated with young plasma.

Figure 2 shows the discrimination of novel objects, also known as novel object recognition (NOR), a familiar and valid form of cognitive memory.

Discrimination index (DI) values in the NOR test of the CO group and sham group decreased significantly compared to the CY group (F (10, 60) = 100.344; *p* = 0.000), (*p* < 0.001). In addition, ovariectomy alone or in combination with Alzheimer’s disease significantly reduced DI in the OVX and OVX + AD groups compared to the CO and sham groups *(p* < 0.001). Exercise and plasma administration significantly increased DI (*p* < 0.001) compared to the OVX and OVX + AD groups (*p* < 0.001).

### 3.2. Spatial Learning and Memory

**Figure 3:**
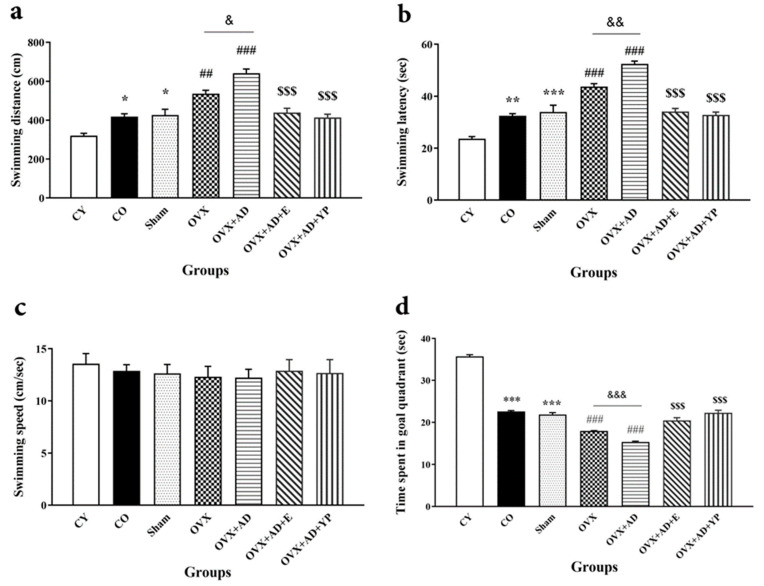
Spatial learning and memory of all experienced groups in Morris water maze (MWM) test. (**a**) Swimming path length. (**b**) Delay time of discovery and jump on the unseen platform. (**c**) Speed of swimming. (**d**) Probe trial for memory retention. Data are accessible as mean ± SEM. and analyzed using repeated measures one-way ANOVA followed by Bonferroni’s and Tukey’s post hoc tests (n = 10), correspondingly. * *p <* 0.05, ** *p <* 0.01 and *** *p <* 0.001 vs. CY group, ## *p <* 0.01 and ### *p <* 0.001 vs. CO and sham groups. $$$ *p <* 0.001 vs. OVX + AD group. & *p* < 0.05, && *p* < 0.01 and &&& *p* < 0.001 vs. OVX group. CY; control young, CO; control old, OVX; ovariectomized, OVX + AD; ovariectomized with Alzheimer’s disease, OVX + AD + E; ovariectomized with Alzheimer’s disease treated with estradiol, OVX + AD + YP; ovariectomized with Alzheimer’s treated with young plasma.

Figure 3 demonstrates the spatial learning and memory for each group with experience using the Morris water maze (MWM) test. Figure 3a; swimming path length (F (10, 60) = 1637.812; *p* = 0.000), Figure 3b; delay period of the discovery of the hidden platform (F (10, 60) = 44.974; *p* = 0.000), Figure 3c; speed of swimming (F (10, 60) = 1.283; *p* = 0.290), and 3d; probe trial for memory retention (F (10, 60) = 236.131; *p* = 0.000).

As revealed in Figure 3a,b, spatial learning was increased significantly for all groups in the second session (second day) of acquisition (*p <* 0.0001). While swimming path and latency time to find the hidden platform was increased significantly in the CO and sham groups compared to CY (*p* < 0.001), there was an increase in the OVX and OVX + AD groups compared to the CO and sham groups (*p* < 0.001).

As shown in Figure 3c, the CO and sham groups spent less time performing recall as a memory retrieval in the target quadrant than CY (*p* < 0.001), and this time was significantly reduced in OVX and OVX + AD compared to the CO and sham groups, respectively, (*p* < 0.001).

The OVX + AD + E and OVX + AD + YP groups spent more time in the target quadrant of the probe trial compared to OVX + AD, and this difference was statistically significant (*p* < 0.001). There were no significant differences between groups in the observation platform and the fast-swimming test *(p* > 0.05).

### 3.3. Passive Avoidance Learning (PAL) Test

**Figure 4:**
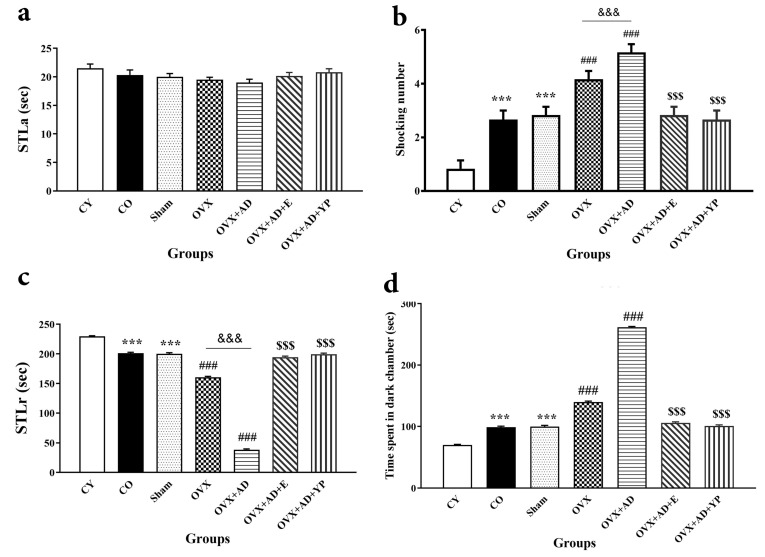
Passive avoidance learning (PAL) tests for all investigational groups. (**a**) Primary step-through latency (STLa or initial latency [IL]). (**b**) Shocking number for complete learning. (**c**) Step-through latency (STLr) to assess memory retention). (**d**) Time spent in the dark after the electrical shock. Data presented as mean ± SEM and analyzed using one-way ANOVA followed Tukey’s post hoc tests (n = 10), respectively. *** *p <* 0.001 vs. CY group, ### *p <* 0.001 vs. CO. and sham groups. $$$ *p <* 0.001 vs. OVX + AD groups. &&& *p <* 0.001 vs. OVX. CY; control young, CO; control old, OVX; ovariectomized, OVX + AD; ovariectomized with Alzheimer’s disease, OVX + AD + E; ovariectomized with Alzheimer’s disease treated with estradiol, OVX + AD + YP; ovariectomized with Alzheimer’s treated with young plasma.

Figure 4 shows passive avoidance learning and memory in the shuttle box device: (a) primary learning as step-through latency (STLa) or initial latency (IL). (b) Shocking number for carrying out learning (F (10, 60) = 18.520; *p* = 0.000). (c) Step-through latency at 24 h after STLa as memory retrieval (F (10, 60) = 1579.383; *p* = 0.000) and (d) time spent in the dark section after STLr as memory perseverance (F (10, 60) = 1579.383; *p* = 0.000).

Statistical analysis confirmed that STLa was not meaningfully different between all experienced groups (F (10, 60) = 1.622; *p* = 0.170, Figure 4a).

In addition, as shown in Figure 4b, the shocking number increased significantly in the CO and sham groups compared to CY (*p <* 0.001), meaningfully augmented in OVX and OVX + AD compared to the CO and sham groups (*p <* 0.001). It decreased significantly in the OVX + AD + E and OVX + AD + YP groups compared to the OVX and OVX + AD groups (*p <* 0.001).

According to the data shown in Figure 4c, there was a significant reduction in STLr in the CO and sham groups compared with the CY group (*p <* 0.001). The STLr significantly deteriorated in OVX and OVX + AD compared to the CO and sham groups (*p <* 0.001), whereas in OVX + AD + E and OVX + AD + YP, STLr was significantly greater compared with OVX + AD amplification (*p* < 0.001).

Given the data revealed in Figure 4d, the TDC of all experimental groups increased significantly compared with the CY group (*p* < 0.001). After comparison between groups, the TDC was significantly increased in OVX and OVX + AD compared to the CO and sham groups (*p <* 0.001). After a comparative analysis, the OVX group showed a significant increase in TDC compared to the OVX group (*p <* 0.001). In contrast, treatment with young plasma and E2 resulted in a significant decrease in TDC compared to the OVX and OVX + AD groups (*p* < 0.001).

### 3.4. Hippocampal Oxidative Stress Markers

**Figure 5:**
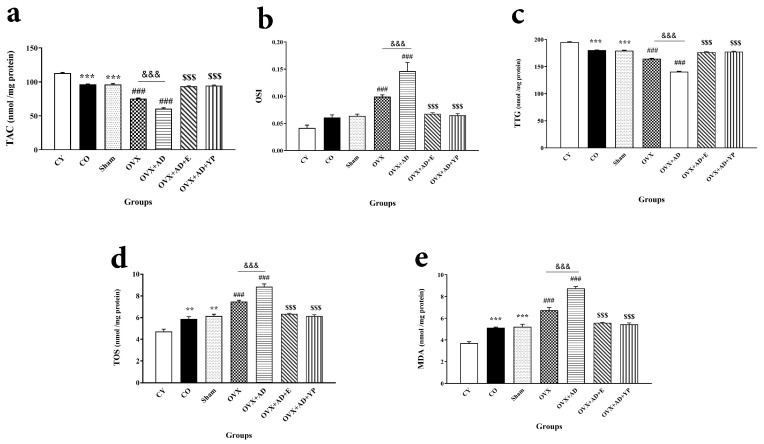
The amount of oxidative stress markers in the hippocampal tissue of considered animals. (**a**) Total antioxidant capacity (TAC). (**b**) Total thiol group (TTG). (**c**) Total oxidant status (TOS). (**d**) Oxidative stress index (OSI). (**e**) Malondialdehyde (MDA). Data are presented as mean ± SEM and analyzed using one-way ANOVA followed by Tukey’s post hoc tests (n = 10), respectively. ** *p <* 0.01 and *** *p <* 0.001 vs. CY. group, ### *p <* 0.001 vs. CO. and sham groups. $$$ *p <* 0.001 vs. OVX + AD groups. &&& *p <* 0.001 vs. OVX. CY; control young, CO; control old, OVX; ovariectomized, OVX + AD: ovariectomized with Alzheimer’s disease, OVX + AD + E; ovariectomized with Alzheimer’s disease treated with estradiol, OVX + AD + YP: ovariectomized with Alzheimer’s treated with young plasma.

As revealed in Figure 5, statistical analysis of TAC (F (6, 49) = 430.966; *p* = 0.000), TTG (F (6, 49) = 504.226; *p* = 0.000), OSI (F (6, 49) = 152.694; *p* = 0.000), TOS (F (6, 49) = 56.123; *p* = 0.000), and MDA (F (6, 49) = 90.762; *p* = 0.000) levels confirmed a notable alteration between the investigational groups.

As shown in Figure 5a,b, the levels of TAC and TTG in the hippocampus of aged rats (sham and CO) were decreased significantly compared to the CY group (*p <* 0.001). These factors’ levels in the OVX and OVX + AD groups were significantly lower than those in the CO and OVX groups, respectively (*p <* 0.001, *p <* 0.001). Treatment with E2 and young plasma caused an increase in TAC and TTG in the hippocampus of aged rats (sham and CO) compared with OVX + AD (*p* < 0.001).

In addition, as shown in Figure 5c,d, analysis of the data displayed that the TOS and OSI levels in the hippocampal tissue of the sham and CO groups were significantly increased compared to those in the CY (*p <* 0.001). Their levels were significantly increased in OVX compared to the CO group (*p* < 0.001).

In OVX + AD, TOS and OSI were improved when analyzed in the OVX group (*p* < 0.001), although treatment of OVX + AD rats with E2 and young plasma meaningfully amplified them (*p* < 0.001).

Figure 5d shows that the MDA level was significantly increased in the hippocampal tissue of the sham and CO groups compared with CY (*p* < 0.001). Its level increased in OVX meaningfully compared with the CO group (*p* < 0.001) and also increased in OVX + AD compared to the OVX group (*p* < 0.001). Treatment of the OVX + AD group with E2 or young plasma meaningfully reversed its level (*p* < 0.001).

### 3.5. Expression of BDNF, p-CREB/CREB, and SIRT-1, miR-134a in the Hippocampus

**Figure 6:**
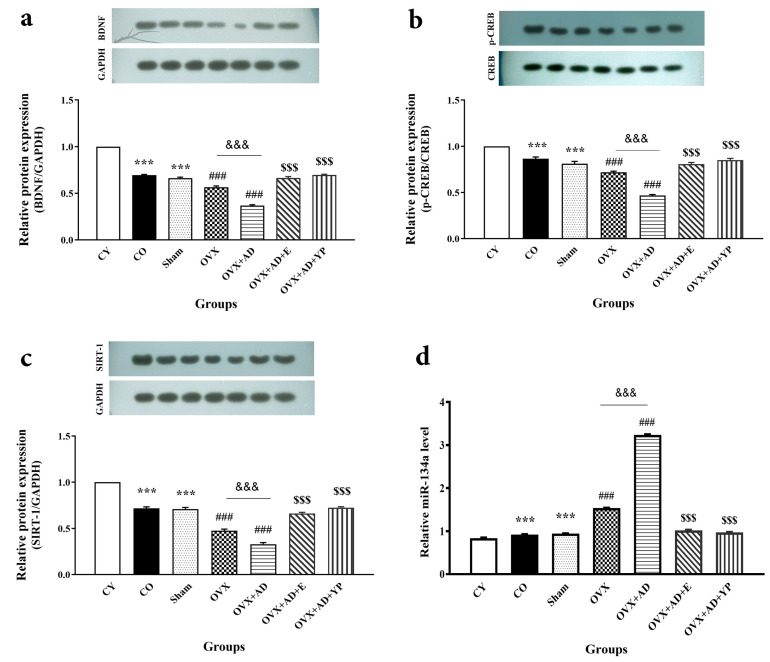
The appearance levels of BDNF, p-CREB/CREB, SIRT-1 protein, and miR-134a expression in the hippocampal tissue of the studied animals. (**a**) Brain-derived neurotrophic factor (BDNF). (**b**) cAMP-responsive element-binding protein (CREB). (**c**) Sirtuin-1 (SIRT-1) protein. (**d**) microRNA-134a (miR-134a). Data are obtainable as mean ± SEM. and analyzed using one-way ANOVA followed by Tukey’s post hoc tests (n = 6), respectively. *** *p <* 0.001 vs. CY group, ### *p <* 0.001 vs. CO. and sham groups. $$$ *p <* 0.001 vs. OVX + AD groups. &&& *p <* 0.001 vs. OVX. CY; control young, CO; control old, OVX; ovariectomized, OVX + AD; ovariectomized with Alzheimer’s disease, OVX + AD + E+ ovariectomized with Alzheimer’s disease treated with estradiol, OVX + AD + YP; ovariectomized with Alzheimer’s treated with young plasma.

As shown in Figure 6, in valuation with the CY group, the other tested groups showed a substantial difference in BDNF expression (F (6, 49) = 370.431; *p* = 0.000, Figure 6), p-CREB/CREB (F (6, 49) = 98.555; *p* = 0.000), SIRT-1 (F (6, 49) = 225.186; *p* = 0.000), and miR-134a (F (6, 49) = 9237.514; *p* = 0.000).

Figure 6a–c shows the results of western blot analysis of protein expression changes in hippocampal tissue. It was determined that the representative value of the molecules was lower in the sham and CO groups than in the CY group (*p <* 0.001). The OVX and OVX + AD groups had remarkable decreases in the expression of BDNF, p-CREB/CREB, and SIRT-1 proteins compared with CO (*p <* 0.001). Treatment of OVX + AD with estradiol or young plasma significantly reversed these effects (*p <* 0.001).

Additionally, as shown in Figure 6d, miR-134a levels were significantly increased in the sham and CO groups compared to CY (*p* < 0.001). Its expression significantly increased in OVX and especially OVX + AD (with greater severity) (*p* < 0.001), and treatment with E2 or young plasma meaningly reversed this increase (*p* < 0.001).

### 3.6. Histopathological Study

**Figure 7:**
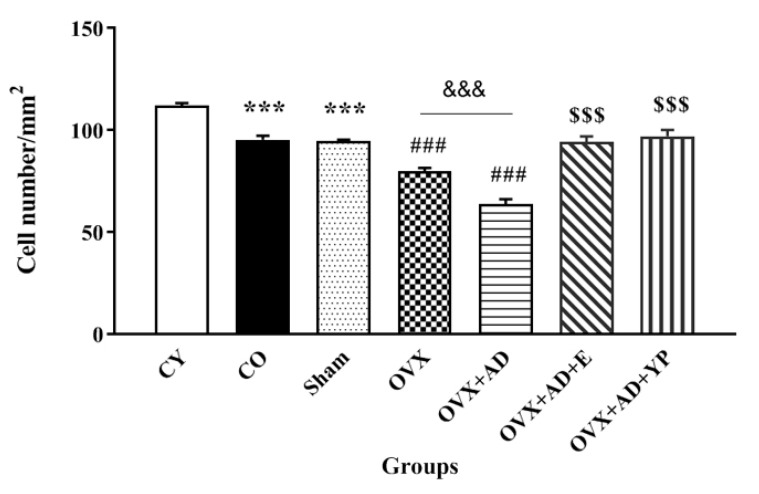

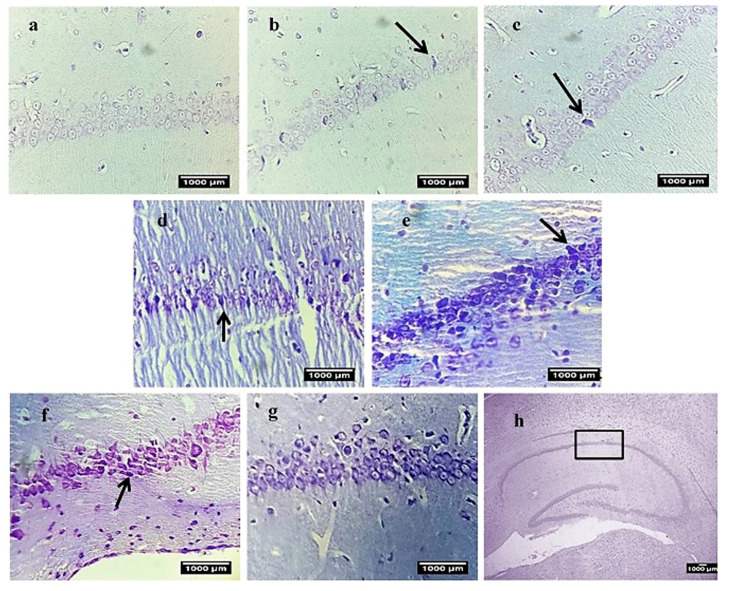
The histological investigation of cell density of hippocampus tissue with Nissl staining and magnification of 400× (Scale bar = 1000 μm) of considered animals. (**a**) CY; control young group, (**b**) CO; control old group, (**c**) sham group, (**d**) OVX; ovariectomized group, (**e**) OVX.AD; ovariectomized group with Alzheimer’s disease. (**f**) OVX.AD.E2; the ovariectomized with Alzheimer’s disease treated with estradiol, (**g**) OVX.AD.YP; the ovariectomized with Alzheimer’s disease and received young plasma, (**h**) CA1 area in the hippocampus of group OVX.AD.YP with an amplification of 100 X (Scale bar = 1000 μm). The black arrows show necrotic nerve cells. The cell density of hippocampus tissue in all experimental groups. Data are obtainable as mean ± SEM and analyzed using one-way ANOVA followed by Bonferroni’s and Tukey’s post hoc tests (n = 4), respectively. *** *p <* 0.001 vs. CY group, ### *p <* 0.001 vs. CO. and sham groups. $$$ *p <* 0.001 vs. OVX + AD groups. &&& *p <* 0.001 vs. OVX. CY; control young, CO; control old, OVX; ovariectomized, OVX + AD; ovariectomized with Alzheimer’s disease, OVX + AD + E; ovariectomized with Alzheimer’s disease treated with estradiol, OVX + AD + YP; ovariectomized with Alzheimer’s treated with young plasma.

The Nissl body contains many rough endoplasmic reticulum and free ribosomes, which synthesize proteins. Therefore, the number of Nissl bodies can be used as an indicator of nerve cell viability. The number of undamaged neurons in the hippocampus of sham and CO groups was lesser than that in the CY (*p <* 0.001, Figure 7a–c). In comparison with the sham and CO groups, there was a significant reduction in the number of positive neurons in the hippocampus of the OVX and OVX + AD groups (*p <* 0.01 and *p <* 0.001, respectively, Figure 7d–e). In contrast, the cell mass was augmented in the OVX + AD + E (*p <* 0.001, Figure 7f) and OVX + AD + YP groups compared with that in the OVX + AD group (Figure 7g, *p <* 0.001). The density of intact neurons in the hippocampal CA1 area of the OVX + AD + YP group is shown in Figure 7h.

## 4. Discussion

In this study, we have reported the effects of young plasma on learning and memory, oxidative stress, miR-134a, BDNF, CREB, and SIRT-1 expression, and the histopathology of the hippocampus in ovariectomized aged rats with Alzheimer’s. This study aimed to compare the cognition, oxidative stress markers, and miR-134 expression in control young and old rats. We also compared the therapeutic potential of estradiol (E2) with young plasma in the treatment of cognitive disorders after the inactivation of ovaries, as well as with estrogen deficiency and neurodegenerative disorders.

The important results were as follows:

1. In the rat model of Alzheimer’s disease, the expression of miR-134a increases with age and estrogen deficiency, followed by a decrease in the expression of proteins such as BDNF, CREB, and SIRT-1 in hippocampal samples.
2. In the aged ovariectomized Alzheimer’s disease rat model, YPT could reduce the negative effects of the hippocampus as much as or more than ERT, improve cognitive behavioral function, and improve the level of antioxidant factors in the hippocampal tissue. Also, cell density was improved in the CA1 area of the hippocampus.

Age-related changes are associated with AD and other neurodegenerative diseases and impair brain function [2]. Ovarian steroids can support many cognitive and neurologic functions [4]. Estrogen deficiency may increase the risk of AD, and estrogen replacement therapy (ERT) has been reported to delay the onset of neurodegenerative disorders in women [39]. In contrast, starting estrogen over age 65 is associated with an increased risk of dementia, and HRT did not have a helpful effect on memory improvement. It is controversial whether it has a neuroprotective effect. ERT is known to increase blood flow to the internal organs of the brain, including the hippocampus, which plays an important role in human memory [40].

The hippocampus, one of the brain areas intricate in memory stabilization, is particularly susceptible to oxidative stress resulting in neuronal death and AD [5]. The decreased expression of several overexpressed brain factors in the hippocampus, such as brain-derived neurotrophic factor (BDNF), cAMP-responsive element-binding protein (CREB), and sirtuin-1 (SIRT-1) protein, is complicated in this vulnerability [10]. On the other hand, due to an estrogen-responsive element in the BDNF gene and the role of estrogen in the up-regulation of SIRT-1 expression and activity, estrogen deficiency is a possible cause of impairment in older postmenopausal women.

Therefore, in general, SIRT-1 is important in brain functioning and neurological diseases [41]. SIRT-1 regulates synaptic plasticity and memory formation through microRNA-mediated mechanisms. These effects are mediated by post-transcriptional regulation of cAMP response binding protein (CREB) expression by the brain-specific microRNA miR-134 [42]. MicroRNAs are small, well-regulated, non-coding RNA molecules that play a role in regulating gene expression and help cells regulate the types and amounts of proteins they produce [43]. Overexpression of miR-134 in the CA1 region also causes severe impairment of long-term memory [44]. SIRT-1 usually leads to the increased expression of CREB and brain-derived neurotrophic factor (BDNF) by dropping the expression of miR-134, thereby growing synaptic plasticity. Moreover, SIRT-1 is involved in a variety of complex processes relevant to aging-associated neuronal degeneration and cognitive decline [28] and aging, including the regulation of oxidative stress [44].

On the other hand, several mechanisms are involved in the existence of cognitive disorders caused by estrogen deficiency and/or aging. Decreased SIRT-1 expression is one of the most important factors in this disease [8].

Hormone therapy (HRT), especially estradiol therapy, is associated with some side effects, including cancer and heart disease, leading to interventions such as juvenile plasma therapy (YPT) as a replacement for HRT. We compared the effects of two treatments on hippocampal cognitive impairment in an ovariectomized rat model of Alzheimer’s disease. Importantly, drugs targeting Aβ fail to restore memory loss in Alzheimer’s patients. In contrast, Heterochronic parabiosis and the administration of juvenile plasma significantly increased levels of amyloid β-protein precursors without reducing the Aβ burden in mice [45]. There are some studies on the effects of young blood transfusions on neurogenesis and cognitive functions in AD models [46]. However, it is unclear whether these changes in blood plasma contribute to brain function in AD models [29]. This study shows that juvenile plasma can reverse cognitive deficits in aged, ovariectomized rats in an AD model. Taken together, previous data suggest that exposure to juvenile blood prevents aging in the hippocampus [22,47]. Aging phenotypes including age-related changes to the brain may potentially be counteracted by “pro-youth” factors from younger individuals [20].

Behavioral findings in this study showed that ovariectomized aged rats with dementia like Alzheimer’s disease have impairments in novel object recognition and discrimination index (DI), spatial memory, and training avoidance. However, treatment with estrogen therapy or youth plasma therapy can improve these conditions. These results are consistent with other similar studies where estrogen deficiency or aging produces similar effects, and the up-regulation of juvenile plasma [48] or E2 [49] is able to resolve these cognitive disorders.

In the present study, the expression level of SIRT-1 in old ovariectomized and/or Alzheimer’s rats decreased significantly, but estrogen and estrogen with young plasma therapies were able to increase their expression level again. The results of this study are in line with other studies showing that SIRT-1 deficiency causes learning disabilities.

Additionally, CREB and BDNF are two genes that have serious functions in synaptic plasticity and altering synapse formation [50]. In SIRT-1 deficiency, both hippocampal BDNF mRNA and protein levels are reduced. CREB binds to multiple BDNF promoters and plays an important role in regulating the activity of BDNF expression. The local binding of CREB to specific BDNF promoters reduces SIRT-1 deficiency in the hippocampus [51]. In our study, the expression of BDNF and CREB significantly decreased in the OVX and OVX + AD groups compared to CY and CO and meaningfully re-established with two estrogen and estrogen combined with YPT therapies. In addition to the behavioral changes in this study, these results are similar to other clinical consequences of estradiol [52], but also the clinical consequences of juvenile plasma injection in the improvement of the expression of CREB and BDNF values. An important mechanism involved in the expression levels of BDNF and CREB is microRNA, which is expressed at high levels in the brain and has been reported to play a positive role in many brain dysfunctions and disorders [40]. MiR-134 is an important microRNA that is specifically expressed in the brain and has been shown to negatively regulate dendritic spines in vitro. MiR-134 directly binds to and inhibits CREB mRNA [44]. Therefore, the increased expression of miR-134 may be accompanied by decreased CREB and BDNF and lead to dementia [44]. In the present study, as explained, the expression levels of miR-134 in OVX and OVX + AD rats were increased significantly, but treatment with estrogen or estrogen combined with YPT was able to neutralize these changes and restore their expression. Similarly, these results are consistent with the results of other studies regarding the therapeutic effects of estradiol [39] but this is the first study to report a treatment in juvenile plasma. Data indicate that the memory and synaptic plasticity defects observed in SIRT-1 deficiency are mainly due to the up-regulation of miR-134 and the resulting repression of miR-134 target genes such as CREB. SIRT-1 normally restricts the expression of miR-134 and, upon SIRT-1 loss-of-function, higher levels of miR-134 negatively regulate synaptic plasticity via the translational inhibition of important plasticity proteins such as CREB, which subsequently mediates the numerous synaptic plasticity impairments that result from SIRT-1 loss-of-function.

As mentioned previously, SIRT-1 plays an important role in the regulation of stress, as well as in regulating the expression of miR-134 and the subsequent expression of CREB and BDNF [19]. Antioxidant and redox signaling (ARS) processes are controlled by important molecules that regulate intracellular antioxidants, reactive oxygen species (ROS) or reactive nitrogen species (RNS), and/or oxidative stress. Disagreement between these molecules can affect cell function and cause disease [21]. Sirtuins such as SIRT-1 are important regulators of ARS in cells. It appears that cellular oxidative stress may cause the dysregulation of normal SIRT-1 function. A 2009 study showed that H2O2-induced oxidative stress reduces SIRT-1.

Additionally, treatment with the SIRT-1 activator resveratrol prevented H2O2-induced cell death, reduced cell proliferation, and inhibited senescence. Alternatively, the SIRT-1 inhibitors sirtuin-1 and nicotinamide were found to increase H2O2-induced cell death. Taken together, these data suggest that SIRT-1 is an important factor in the protection of oxidative damage by various mechanisms [19]. Compared to other studies, our study also showed a decrease in the results of antioxidant markers such as total antioxidant capacity (TAC) and total thiol group (TTG), but oxidative stress markers such as malondialdehyde (MDA) did not significantly affect the overall oxidative status. The total oxidant status (TOS) and oxidative stress index (OSI) were significantly increased in the OVX and OVX + AD groups. Estrogen and YPT treatment can restore normal results. The results in this section are similar to previous measurements, correspond to changes in behavior and biochemical parameters, and are consistent with the results of other studies on the treatment status of estradiol [53] and YPT [54]. However, the effects of YPT on oxidative stress in an ovariectomized aged rat model with or without Alzheimer’s disease are reported for the first time.

In addition to behavioral and biochemical tests, a histopathological study was performed on hippocampal tissue. Nissl bodies contain many rough endoplasmic reticulum and free ribosomes that produce proteins. Therefore, the number of Nissl bodies can be used as a marker of neuronal cell viability. According to the results, estrogen deficiency and Aβ caused more deaths in the hippocampal CA1 region of the rats in the OVX and OVX + AD groups than in the rats in the sham group. However, neuronal damage in the treated group of rats was reduced with juvenile plasma or estradiol treatment; this indicates that estradiol and YPT can protect against neuronal damage in OVX and/or Aβ-induced AD rats.

As previously mentioned, this study is the first to investigate the experimental effect of plasma administration on cognitive impairment in adolescents and its associated characteristics. However, estrogen therapy has produced conflicting results. Ovarian steroids affect many cognitive and neurological processes that contribute to cognitive function [55]. As shown in this and other studies, estrogen deficiency can increase the risk of AD, but ERT has been reported to delay the onset of neurodegenerative disease in women [56]. In contrast, taking estrogen over the age of 65 increases the risk of dementia [51], and HRT does not affect the improvement of memory [39]. Additionally, hormonal therapy (HRT), especially estradiol therapy, is often associated with many side effects such as cancer and heart disease, so HPT may be necessary for hormonal therapy.

## 5. Conclusions

According to the results, estrogen deficiency and/or AD caused oxidative stress effects, decreased levels of BDNF, p-CREB, and SIRT-1 proteins, and increased miR-134a expression in the hippocampus. Our results showed that YPT improves cognitive impairment and CA1 hippocampal cell density as much as or more than ERT by altering biochemical and molecular signaling factors. This study, in line with other work, shows that young plasma therapy may be superior to hormone therapy in delaying or even preventing sequelae of aging such as menopause or Alzheimer’s disease. Therefore, more research on other variables and animal models is needed before the results of this study can be generalized to humans.

## Data Availability

All data included in this study are available upon request from the corresponding author. The data are not publicly available due to institutional copyright policy.

## References

[1] Rao Y.L., Ganaraja B., Murlimanju B., Joy T., Krishnamurthy A., Agrawal A. (2022). Hippocampus and its involvement in Alzheimer’s disease: A review. 3 Biotech.

[2] Conde D.M., Verdade R.C., Valadares A.L., Mella L.F., Pedro A.O., Costa-Paiva L. (2021). Menopause and cognitive impairment: A narrative review of current knowledge. World J. Psychiatry.

[3] Mosconi L., Berti V., Dyke J., Schelbaum E., Jett S., Loughlin L., Jang G., Rahman A., Hristov H., Pahlajani S. (2021). Menopause impacts human brain structure, connectivity, energy metabolism, and amyloid-beta deposition. Sci. Rep..

[4] Li J., Gibbs R.B. (2019). Detection of estradiol in rat brain tissues: Contribution of local versus systemic production. Psychoneuroendocrinology.

[5] Ionescu-Tucker A., Cotman C.W. (2021). Emerging roles of oxidative stress in brain aging and Alzheimer’s disease. Neurobiol. Aging.

[6] You Y., Liang W. (2023). SIRT1 and SIRT6: The role in aging-related diseases. Biochim. Biophys. Acta Mol. Basis Dis..

[7] Herskovits A.Z., Guarente L. (2014). SIRT1 in neurodevelopment and brain senescence. Neuron.

[8] Lu B., Nagappan G., Lu Y. (2014). BDNF and synaptic plasticity, cognitive function, and dysfunction. Neurotrophic Factors.

[9] Komori T., Okamura K., Ikehara M., Yamamuro K., Endo N., Okumura K., Yamauchi T., Ikawa D., Ouji-Sageshima N., Toritsuka M. (2024). Brain-derived neurotrophic factor from microglia regulates neuronal development in the medial prefrontal cortex and its associated social behavior. Mol. Psychiatry.

[10] Shen J., Xu L., Qu C., Sun H., Zhang J. (2018). Resveratrol prevents cognitive deficits induced by chronic unpredictable mild stress: Sirt1/miR-134 signalling pathway regulates CREB/BDNF expression in hippocampus in vivo and in vitro. Behav. Brain Res..

[11] Coolen M., Bally-Cuif L. (2015). MicroRNAs in brain development. MicroRNA in Regenerative Medicine.

[12] Rashidi S.K., Kalirad A., Rafie S., Behzad E., Dezfouli M.A. (2023). The role of microRNAs in neurobiology and pathophysiology of the hippocampus. Front. Mol. Neurosci..

[13] Eyileten C., Sharif L., Wicik Z., Jakubik D., Jarosz-Popek J., Soplinska A., Postula M., Czlonkowska A., Kaplon-Cieslicka A., Mirowska-Guzel D. (2021). The relation of the brain-derived neurotrophic factor with microRNAs in neurodegenerative diseases and ischemic stroke. Mol. Neurobiol..

[14] Wang I.-F., Ho P.-C., Tsai K.-J. (2022). MicroRNAs in learning and memory and their impact on Alzheimer’s disease. Biomedicines.

[15] Association A.s., Thies W., Bleiler L. (2013). Alzheimer’s disease facts and figures. Alzheimer’s Dement..

[16] Chen C., Zhou M., Ge Y., Wang X. (2020). SIRT1 and aging related signaling pathways. Mech. Ageing Dev..

[17] Javani G., Alihemmati A., Habibi P., Yousefi H., Karimi P., Ebraheimi V., Ahmadiasl N. (2019). The effects of genistein on renal oxidative stress and inflammation of ovariectomized rats. Jundishapur J. Nat. Pharm. Prod..

[18] Conboy I.M., Rando T.A. (2012). Heterochronic parabiosis for the study of the effects of aging on stem cells and their niches. Cell Cycle.

[19] Eggel A., Wyss-Coray T. (2014). Parabiosis for the study of age-related chronic disease. Swiss Med. Wkly..

[20] Villeda S.A., Plambeck K.E., Middeldorp J., Castellano J.M., Mosher K.I., Luo J., Smith L.K., Bieri G., Lin K., Berdnik D. (2014). Young blood reverses age-related impairments in cognitive function and synaptic plasticity in mice. Nat. Med..

[21] Ashapkin V.V., Kutueva L.I., Vanyushin B.F. (2020). The effects of parabiosis on aging and age-related diseases. Rev. New Drug Targets Age-Relat. Disord..

[22] Hernandez C.M., Barkey R.E., Craven K.M., Pedemonte K.A., Alisantosa B., Sanchez J.O., Flinn J.M. (2023). Transfusion with blood plasma from young mice affects rTg4510 transgenic tau mice modeling of Alzheimer’s disease. Brain Sci..

[23] Chen X., Luo Y., Zhu Q., Zhang J., Huang H., Kan Y., Li D., Xu M., Liu S., Li J. (2024). Small extracellular vesicles from young plasma reverse age-related functional declines by improving mitochondrial energy metabolism. Nat. Aging.

[24] Williams S., Wakisaka A., Zeng Q., Barnes J., Martin G., Wechter W., Liang C. (1996). Minocycline prevents the decrease in bone mineral density and trabecular bone in ovariectomized aged rats. Bone.

[25] Babaei P., Mehdizadeh R., Ansar M.M., Damirchi A. (2010). Effects of ovariectomy and estrogen replacement therapy on visceral adipose tissue and serum adiponectin levels in rats. Menopause Int..

[26] Liu A., Guo E., Yang J., Yang Y., Liu S., Jiang X., Hu Q., Dirsch O., Dahmen U., Zhang C. (2018). Young plasma reverses age-dependent alterations in hepatic function through the restoration of autophagy. Aging Cell.

[27] Habibi P., Alihemmati A., Alipour M., Yousefi H., Andalib S., Ahmadiasl N. (2016). Effects of exercise on miR-29 and IGF-1 expression and lipid profile in the heart of ovariectomized rat. Acta Endocrinol..

[28] Kashani M.S., Tavirani M.R., Talaei S.A., Salami M. (2011). Aqueous extract of lavender (*Lavandula angustifolia*) improves the spatial performance of a rat model of Alzheimer’s disease. Neurosci. Bull..

[29] Zhao Y., Qian R., Zhang J., Liu F., Iqbal K., Dai C.-L., Gong C.-X. (2020). Young blood plasma reduces Alzheimer’s disease-like brain pathologies and ameliorates cognitive impairment in 3× Tg-AD mice. Alzheimer’s Res. Ther..

[30] Almasi A., Zarei M., Raoufi S., Sarihi A., Salehi I., Komaki A., Hashemi-Firouzi N., Shahidi S. (2018). Influence of hippocampal GABA B receptor inhibition on memory in rats with acute β-amyloid toxicity. Metab. Brain Dis..

[31] Lu Y., Dong Y., Tucker D., Wang R., Ahmed M.E., Brann D., Zhang Q. (2017). Treadmill exercise exerts neuroprotection and regulates microglial polarization and oxidative stress in a streptozotocin-induced rat model of sporadic Alzheimer’s disease. J. Alzheimer’s Dis..

[32] Babri S., Amani M., Mohaddes G., Alihemmati A., Ebrahimi H. (2012). Protective effects of troxerutin on β-amyloid (1-42)-induced impairments of spatial learning and memory in rats. Neurophysiology.

[33] Habibi P., Babri S., Ahmadiasl N., Yousefi H. (2017). Effects of genistein and swimming exercise on spatial memory and expression of microRNA 132, BDNF, and IGF-1 genes in the hippocampus of ovariectomized rats. Iran. J. Basic Med. Sci..

[34] Sadeghian R., Shahidi S., Komaki A., Habibi P., Ahmadiasl N., Yousefi H., Daghigh F. (2021). Synergism effect of swimming exercise and genistein on the inflammation, oxidative stress, and VEGF expression in the retina of diabetic-ovariectomized rats. Life Sci..

[35] Benzie I.F., Strain J. (1999). Ferric reducing/antioxidant power assay: Direct measure of total antioxidant activity of biological fluids and modified version for simultaneous measurement of total antioxidant power and ascorbic acid concentration. Methods in Enzymology.

[36] Erel O. (2005). A new automated colorimetric method for measuring total oxidant status. Clin. Biochem..

[37] Mohseni R., Karimi J., Tavilani H., Khodadadi I., Hashemnia M. (2020). Carvacrol downregulates lysyl oxidase expression and ameliorates oxidative stress in the liver of rats with carbon tetrachloride-induced liver fibrosis. Indian J. Clin. Biochem..

[38] Livak K.J., Schmittgen T.D. (2001). Analysis of relative gene expression data using real-time quantitative PCR and the 2^−ΔΔCT^ method. Methods.

[39] Markham J., Pych J., Juraska J. (2002). Ovarian hormone replacement to aged ovariectomized female rats benefits acquisition of the morris water maze. Horm. Behav..

[40] LeBlanc E.S., Janowsky J., Chan B.K., Nelson H.D. (2001). Hormone replacement therapy and cognition: Systematic review and meta-analysis. JAMA.

[41] Mielke M.M., Vemuri P., Rocca W.A. (2014). Clinical epidemiology of Alzheimer’s disease: Assessing sex and gender differences. Clin. Epidemiol..

[42] Mosconi L., Berti V., Quinn C., McHugh P., Petrongolo G., Varsavsky I., Osorio R.S., Pupi A., Vallabhajosula S., Isaacson R.S. (2017). Sex differences in Alzheimer risk: Brain imaging of endocrine vs chronologic aging. Neurology.

[43] Mosconi L., Berti V., Guyara-Quinn C., McHugh P., Petrongolo G., Osorio R.S., Connaughty C., Pupi A., Vallabhajosula S., Isaacson R.S. (2017). Perimenopause and emergence of an Alzheimer’s bioenergetic phenotype in brain and periphery. PLoS ONE.

[44] Ross R.K., Paganini-Hill A., Mack T.M., Henderson B.E. (1989). Cardiovascular benefits of estrogen replacement therapy. Am. J. Obstet. Gynecol..

[45] Middeldorp J., Lehallier B., Villeda S.A., Miedema S.S., Evans E., Czirr E., Zhang H., Luo J., Stan T., Mosher K.I. (2016). Preclinical assessment of young blood plasma for Alzheimer disease. JAMA Neurol..

[46] Ma J., Gao B., Zhang K., Zhang Q., Jia G., Li J., Li C., Yan L.-J., Cai Z. (2019). Circulating factors in young blood as potential therapeutic agents for age-related neurodegenerative and neurovascular diseases. Brain Res. Bull..

[47] Xia E., Xu F., Hu C., Kumal J.P.P., Tang X., Mao D., Li Y., Wu D., Zhang R., Wu S. (2019). Young blood rescues the cognition of Alzheimer’s model mice by restoring the hippocampal cholinergic circuit. Neuroscience.

[48] McKinney B.C., Lin C.-W., Oh H., Tseng G.C., Lewis D.A., Sibille E. (2015). Hypermethylation of BDNF and SST genes in the orbital frontal cortex of older individuals: A putative mechanism for declining gene expression with age. Neuropsychopharmacology.

[49] Carbone D.L., Handa R.J. (2013). Sex and stress hormone influences on the expression and activity of brain-derived neurotrophic factor. Neuroscience.

[50] Guo J.M., Shu H., Wang L., Xu J.J., Niu X.C., Zhang L. (2017). SIRT 1-dependent AMPK pathway in the protection of estrogen against ischemic brain injury. CNS Neurosci. Ther..

[51] Dongare S.S., Gupta S.K., Mathur R., Saxena R., Srivastava S., Nag T.C. (2015). Genistein ameliorates diabetic retinopathy by suppression of oxidative stress, inflammation and angiogenic markers in streptozotocin induced retinal neovascularization in neonatal rats (nSTZ). Investig. Ophthalmol. Vis. Sci..

[52] Gleason C.E., Dowling N.M., Wharton W., Manson J.E., Miller V.M., Atwood C.S., Brinton E.A., Cedars M.I., Lobo R.A., Merriam G.R. (2015). Effects of hormone therapy on cognition and mood in recently postmenopausal women: Findings from the randomized, controlled KEEPS–cognitive and affective study. PLoS Med..

[53] Ruckh J.M., Zhao J.-W., Shadrach J.L., van Wijngaarden P., Rao T.N., Wagers A.J., Franklin R.J. (2012). Rejuvenation of regeneration in the aging central nervous system. Cell Stem Cell.

[54] Tripathi S.S., Kumar R., Arya J.K., Rizvi S.I. (2021). Plasma from Young Rats Injected into Old Rats Induce Antiaging Effects. Rejuvenation Res..

[55] Koebele S.V., Nishimura K.J., Bimonte-Nelson H.A., Kemmou S., Ortiz J.B., Judd J.M., Conrad C.D. (2020). A long-term cyclic plus tonic regimen of 17β-estradiol improves the ability to handle a high spatial working memory load in ovariectomized middle-aged female rats. Horm. Behav..

[56] He Q., Luo Y., Lv F., Xiao Q., Chao F., Qiu X., Zhang L., Gao Y., Xiu Y., Huang C. (2018). Effects of estrogen replacement therapy on the myelin sheath ultrastructure of myelinated fibers in the white matter of middle-aged ovariectomized rats. J. Comp. Neurol..

